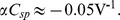# Modeling Electrically Active Viscoelastic Membranes

**DOI:** 10.1371/journal.pone.0037667

**Published:** 2012-05-31

**Authors:** Sitikantha Roy, William E. Brownell, Alexander A. Spector

**Affiliations:** 1 Department of Biomedical Engineering, Johns Hopkins University, Baltimore, Maryland, United States of America; 2 Bobby R. Alford Department of Otolaryngology, Head & Neck Surgery, Baylor College of Medicine, Houston, Texas, United States of America; University of Arizona, United States of America

## Abstract

The membrane protein prestin is native to the cochlear outer hair cell that is crucial to the ear's amplification and frequency selectivity throughout the whole acoustic frequency range. The outer hair cell exhibits interrelated dimensional changes, force generation, and electric charge transfer. Cells transfected with prestin acquire unique active properties similar to those in the native cell that have also been useful in understanding the process. Here we propose a model describing the major electromechanical features of such active membranes. The model derived from thermodynamic principles is in the form of integral relationships between the history of voltage and membrane resultants as independent variables and the charge density and strains as dependent variables. The proposed model is applied to the analysis of an active force produced by the outer hair cell in response to a harmonic electric field. Our analysis reveals the mechanism of the outer hair cell active (isometric) force having an almost constant amplitude and phase up to 80 kHz. We found that the frequency-invariance of the force is a result of interplay between the electrical filtering associated with prestin and power law viscoelasticity of the surrounding membrane. Paradoxically, the membrane viscoelasticity boosts the force balancing the electrical filtering effect. We also consider various modes of electromechanical coupling in membrane with prestin associated with mechanical perturbations in the cell. We consider pressure or strains applied step-wise or at a constant rate and compute the time course of the resulting electric charge. The results obtained here are important for the analysis of electromechanical properties of membranes, cells, and biological materials as well as for a better understanding of the mechanism of hearing and the role of the protein prestin in this mechanism.

## Introduction

The lipid bilayers in biological membranes contain electrical charges and dipoles, and conformational changes of membrane proteins transport extrinsic ions and move intrinsic charged residues [1]. These membrane structures are associated with a variety of modes of electromechanical coupling. For example, conformational changes in proteins are related to gating currents [2], [3] and mechanosensitive channels can be activated, de-activated, or switched from one of the multiple states to another as a result of mechanical factors, such as force, stress, or strain in the surrounding membranes. In cellular membranes, the channels can be also governed by the viscoelastic-like interaction between the plasma membrane and underlying cytoskeleton [4], [5]. Lipid bilayers have curvatures due to the membrane composition and embedded membrane proteins [6], [7] and there is a bidirectional relationship between the membrane polarization and changes in these membrane curvatures (direct and converse flexoeffects) [8]. The mechanical properties of tissues and biological materials depend on the interaction between the intrinsic charges and surrounding tissue components [9]–[11].

The inner ear (cochlea) converts the mechanical sound waves into an electrical signal going to the brain. Electromechanical coupling is a critical feature of the cochlea and it is believed that two modes of such coupling, acting in concert, provide the mammalian ear with sound amplification and sharp frequency selectivity [12]. The first of these two modes is dependent on the ability the cochlear outer hair cell to change its length and (if constrained) to generate an active force in response to changes in the cell membrane potential [13], [14]. The second mode is associated with the bundle of stereocilia atop of this cell. Physiological deflection of this bundle produces a mechanotransduction current, thereby changing the membrane potential of outer hair cells. In addition, the hair cell bundle can also generate an active force [15], [16].

The protein prestin was originally discovered in the outer hair cells of the mammalian cochlea where it is distributed along the cellular plasma membrane [17]. Structurally, the outer hair cell has a pressurized liquid core bounded by a composite, three-layer membrane (wall). The outermost part is the plasma membrane, the cytoskeleton is located below it, and the innermost component is the subsurface cisternae. To better understand the structure and function of this protein, other mammalian cells (e.g., human embryonic kidney [HEK] and Chinese hamster ovary [CHO] cells) were transfected with prestin and used for genetic manipulations. After transfection, these cells acquire the major features of the native outer hair cell [17], [18]. Prestin is critical to electromechanical coupling (electromotility) in the outer hair cell and to active hearing in general [19], [20]. It is believed that prestin undergoes a conformational change during transfer of an electric charge. Prestin's ability to generate electrical charges (current) in response to mechanical factors (e, g., pressure [21], [22] or strain [23]) and to alter the cell length in response to the application of an electric field [13] constitute a piezoelectric-like effect. An earlier piezoelectric relationship [24] described the coupling between the integral active force and the total charge generated by the outer hair cell. This relationship, including its thermodynamically-necessary symmetry, was later confirmed experimentally [25]. Recently, a nonlinear circuit version of the outer hair cell charge-force model was developed [26] and continuum (linear and nonlinear) piezoelectric equations for the outer hair cell composite membrane were proposed [27]–[29]. In these constitutive models of the outer hair cell membrane, the mechanical and electrical parts were considered as purely elastic and purely capacitive, respectively. It has been shown [30], however, that the electric charge transfer by prestin is associated with resistive current as well.

A physical mechanism involving a two-component (capacitive and resistive) charge associated with prestin has recently been proposed, with the resistive component appearing naturally as a result of a delay between the moving charge and the applied electric field. This delay increases with the frequency of the applied field [30]. Also, a recent model of the outer hair cell's power efficiency has included electrical conductivity in the constitutive differential equation describing the electrical properties of the outer hair cell membrane [31]. The frequency effects on both prestin and outer hair cell performance are critical because both are active up to tens of kHz. Thus, a rate- (frequency-) dependent component needs to be incorporated in the mechanical part of the model. In the case of other cell types, it has been shown that the mechanical (viscoelastic) properties have been effectively described by a power law in which the real and imaginary parts of the complex modulus are similar power functions of frequency [32]–[36]. Whether this approach is also effective for the outer hair cell and what the corresponding model parameters are has remained an open question particularly since this cell functions within an extremely broad frequency range of several tens of kHz.

**Figure 1:**
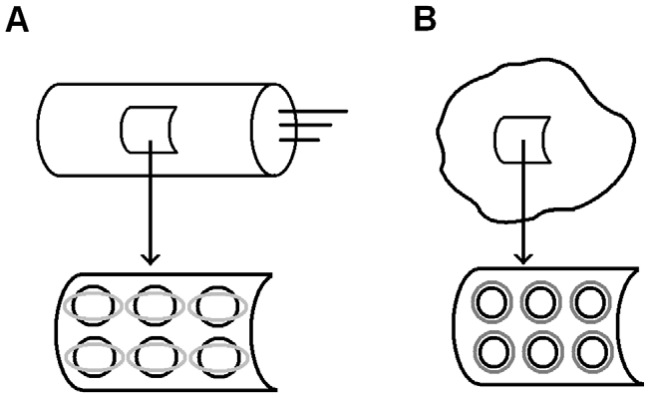
Cellular membranes containing the protein prestin. A) Upper panel: outer hair cell. Lower panel: representative cut from the outer hair cell composite membrane (wall). The molecules of prestin distributed along the membrane (cell) surface undergo conformational changes schematically sketched as black circles transforming into gray ellipses. This elliptical shape is consistent with the active strain being compressive in the circumferential direction and extensive in the longitudinal direction along the cylindrical cell whose wall has anisotropic properties [37]. B) Upper panel: cell transfected with prestin and representative section of its membrane. Lower panel: representative section of its membrane. The molecules of prestin distributed along the membrane (cell) surface undergo conformational changes schematically sketched as black circles transforming into gray circles.

Here we describe the outer hair cell using a cylindrical coordinate system (x_1_, x_2_) with the x_1_-axis directed along the cell. We consider axysimmertic states of the membrane with no bending. We use the strain (ε_1_, ε_2_) and resultants (N_1_, N_2_) as the mechanical characteristics of the membrane. We also use membrane voltage (potential), V, and electric charge per unit area, q, as the electrical characteristics of the membrane. For the simplicity, we treat the case of cells transfected with prestin as spherically symmetric. For both outer hair cells and cells transfected with prestin, we assume that the prestin molecules act mechanically as relatively rigid inclusions in a viscoelastic matrix, and these inclusions change their shape (make conformational changes) in response to changes in the transmembrane potential (Fig. 1). Similar to earlier (quasistatic) models [37], we assume that the total observable strain is the sum of the prestin-associated (active, ε_1_^a^, ε_2_^a^) and matrix-associated (passive, ε_1_^p^, ε_2_^p^) components. A common assumption regarding prestin's function is that this protein's conformational changes are triggered by the transfer of an electric charge, and the active strain results from the accumulation of such molecular-level changes made by the proteins distributed along the plasma membrane.

We propose a constitutive model that generalizes piezoelectric and viscoelastic relationships and includes the main modes of electromechanical coupling in outer hair cells and cells transfected with prestin. The model is derived from first thermodynamic principles and is effective over the whole acoustic frequency range of cell functioning. We apply the developed model to analyze the key characteristics of cells containing membranes with prestin. Our analysis explains the phenomenon of an active force generated by outer hair cell that has an almost constant amplitude and phase up to about 80 kHz. Our model reveals that the observed frequency-invariance of the force is a result of interplay between the electrical filtering properties of prestin and power law viscoelasticity of the surrounding membrane. We also consider other modes of electromechanical coupling in the outer hair cell membrane and membranes of cells with prestin. We found the time course of charge building in response to changes in the mechanical factors, including pressure and strains, and consider different regimes when these factors change step-wise or with a constant rate. The results we have obtained have potential significance for modeling electromechanical coupling (mechanotransduction) in membranes, cells, and tissues and can also contribute to a better understanding of the hearing process and its molecular mechanism.

## Results and Discussion

### Cell Active Force Production throughout Acoustic Frequency Range

**Figure 2:**
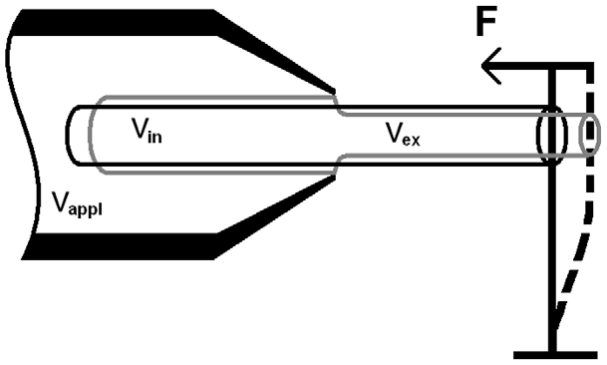
Outer hair cell's active force measurement in the microchamber experiment. The cell is partially inserted into a large pipette (microchamber), and voltage V_app_ is applied to the chamber environment. The voltage in the microchamber results in the membrane potentials, V_in_, and V_ex_, of the included and excluded parts of the cell. These potentials have opposite signs, and the two (included and excluded) parts of the electromotile cell become, respectively, shorter and thicker and longer and thinner. The excluded part pushes the AFM cantilever of a prescribed stiffness, and the generated active force, F, can be estimated based on the displacement of the cantilever.

**Figure 3:**
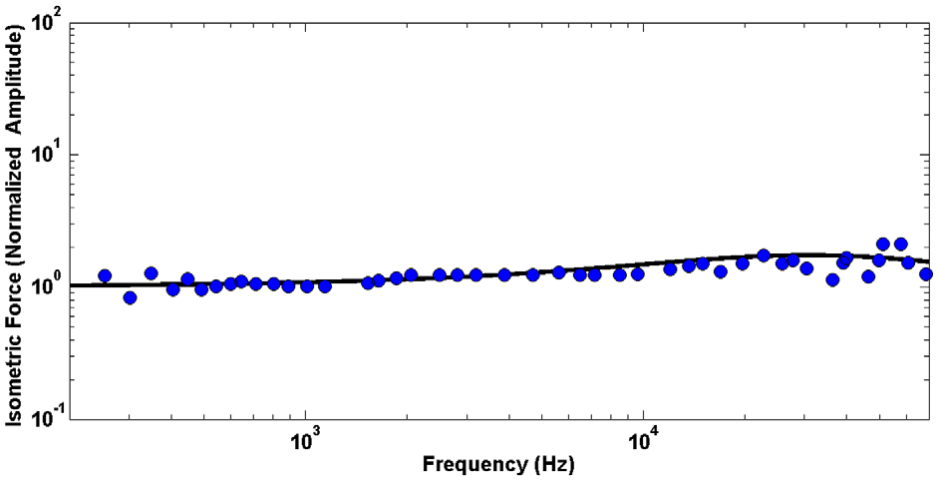
Model application to the analysis of the outer hair cell active force. Circles: experimental data from [38] of the active force amplitude (normalized by its low frequency value). Solid line: current model for the optimal parameters of membrane viscoelasticity, 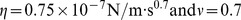.

**Figure 4:**
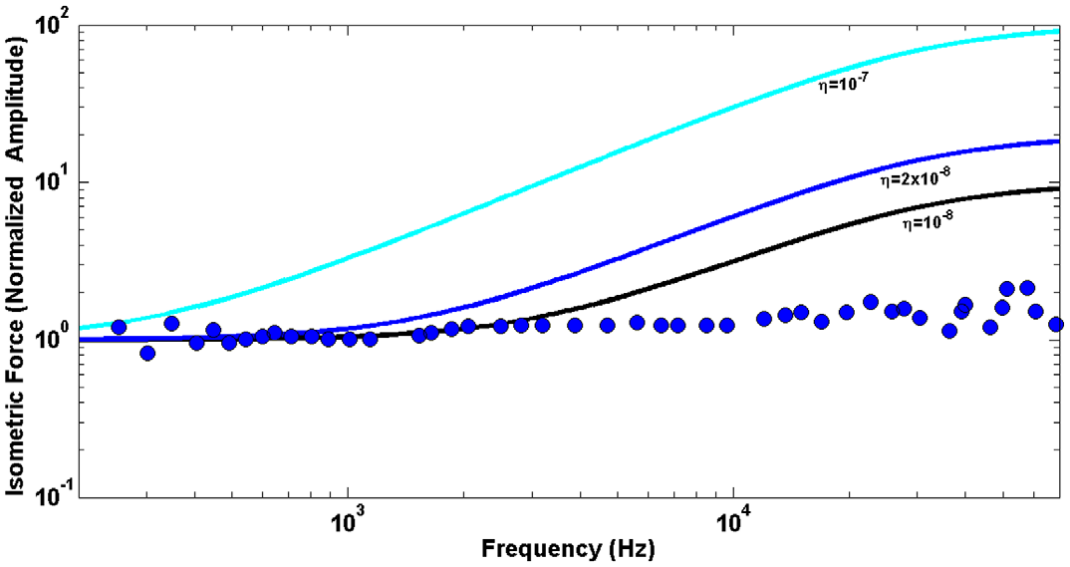
Predictions of Kelvin-Voight models. Predictions of a Kelvin-Voight-like model of membrane viscoelasticity with three different viscosities, 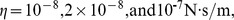 (color lines) vs. the experimental values of the normalized amplitude of the outer hair cell active force (circles) from [38].

**Figure 5:**
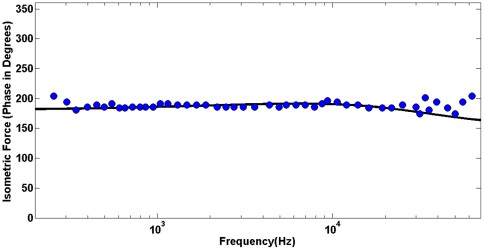
Phase of the outer hair cell active force. Model prediction of the phase of the active force (solid line) vs. experimental data of [38] (circles) for the optimal parameters of membrane viscoelasticity, 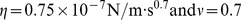.

The active force production by outer hair cells is of primary physiological significance providing the amplification and frequency selectivity of the ear. Thus we start with the application of our model to the analysis of the active force focusing on outer hair cell's remarkable ability to generate a force of almost constant amplitude and phase throughout the whole acoustic frequency range, up to 70–80 kHz. A sketch of the experiment to measure such force is shown in Fig. 2 where the cell is partially included in a large pipette (microchamber) and is stimulated by a harmonic electric field applied to the solution inside the pipette [38] The cell is constrained by a stiff atomic force cantilever whose deflection resulting from the applied electric field is measured. This experimental arrangement simulates the cell condition in vivo where it is constrained by much stiffer cochlear structures. Due to a minimal movement of the highly-constrained cell, the corresponding active force can be interpreted as an isometric force. The derivation of the outer hair cell isometric force from the constitutive equations of our electro-viscoelastic model is given below in the Methods section. First, we analyze the amplitude of the force (Eq. 22). Fig. 3 presents the computed values of this amplitude normalized by its zero-frequency value (black solid line) vs. experimental data of [38] (blue circles). For our analysis here, we choose a frequency range of relatively small variations of the original experimental data in [38] (this variation increases above 80 kHz). We present our results in the same log-log scale that was used for the presentation of the original experiment. In Fig. 3, the computational results are obtained for optimal parameters of the power law of the cell membrane viscoelasticity (see the Methods section),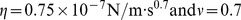, that provide the best fit (least square minimization) of the experimental data. To emphasize a critical role of the power law viscolasticity in our analysis, we also present the active force computed on the basis of a traditional Kelvin-Voight viscoelastic model. Fig. 4 shows the obtained results for three different values of the membrane viscosity, 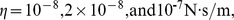 (color lines) vs. the same experimental data as before (blue circles) of [38]. Finally, we analyze the phase of the active force. We obtain the phase of the active force for the same parameters that were estimated as optimal in the above analysis of the force amplitude. The force phase is computed using Eqs. 23 and 17 and presented in Fig. 5 (black line) vs. experimental data of [38] (blue circles).

These results can shed a new light on the mechanics of the active force production by the outer hair cell and cell membranes with prestin. One of the most physiologically significant results coming from the microchamber experiment [38] was the observation that the outer hair cell can produce an active force of almost the same amplitude and phase throughout a broad frequency range up to about 80 kHz. Note that in the case of cells transfected with prestin, the computational results (not included) with the same power law elasticity and prestin-associated time also show an isometric force of almost constant amplitude and phase consistent with experimental data of [18]. One reason for the constant force is a minimal interaction with the surrounding viscous fluid because the cell has no observable strain (movement) under isometric conditions. However, there are at least two additional dissipative mechanisms, the prestin electrical resistivity and membrane viscoelasticity, that might affect the frequency dependence of the active force. An interesting conclusion from our model analysis is that these two mechanisms do not sum but rather balance each other in the process of the active force production. Indeed both the viscoelasticity-related numerator and presin resistivity-determined denominator in Eq. 22 increase with frequency resulting in almost frequency-independent force amplitude. It can intuitively be expected that the viscous properties of the membrane could cause a cut-off type of effect on the active force. The model shows that viscoelasticity actually provides the increase in the active force with frequency, which of course is balanced by a decrease caused by the electrical effect. Similarly, the two mechanisms balance each other, keeping the phase of the active force almost independent of frequency: the prestin-related phase is negative, and the viscoelasticity-related parties positive. Such a feature whereby the viscosity contributes to the active cochlear (outer hair cell) forces and balances the electrical filtering has previously been discussed in [39].

The frequency dependence of the active force is quite sensitive to the parameters of the power law viscoelasticity: for example, Kelvin-Voight-like models with ν = 1 and realistic values of the viscosity, η, result in a significant increase in the force with frequency, which contradicts the experimental data (Fig. 5). Power law viscoelasticity has been shown to be effective in the description of cellular behavior. The resulting viscoelastic parameters differ depending on type of the cell and frequency range (time scale). A variety of cells considered in the frequency range of 1 Hz–1 kHz were described by the law with a power of 0.2 (e.g. [32]). It was later shown that if the frequency range is extended toward lower frequencies, the cell behavior can be described in terms of two powers: one close to 0.33 and the other of about 0.2 for frequency ranges of 10^−3^–10^−1^ Hz and 1 Hz–10^3^ Hz, respectively [33]. In addition, studies of adherent cells developed at their physiological frequency limit (about 1 kHz) showed that these cells follow power law viscoelasticity with an exponent of 0.75 [34]. In some cells, probing the mechanical properties was extended to very high frequencies, and the results confirmed the power law rheology [35]. It has recently been demonstrated [36] that cell rheology can be effectively described by a weak power law for low frequencies and a stronger frequency dependence of the cell shear modulus given by a power of 0.75. The outer hair cell and cells transfected with prestin operate in a frequency range (tens of kilohertz) that was much greater than physiological ranges of other cells. Yet, we show that the power law viscoelasticity can effectively describe the main features of these cells too. The experimental data that we used to extract the viscoelastic parameters of our model are not detailed enough to consider separately a low frequency range like it was done in [36], and we use the same power law throughout all frequencies. The obtained power of 0.7 is close to the upper end of such parameters in previously studied cells.

### Electromechanical Coupling

While the outer hair cell active force is an important outcome of the cell performance in the cochlea, other electromechanical modes are involved too and they shed light on the mechanism of the active force production. Electromechanical coupling is a key feature of the outer hair membrane and other cells acquire similar properties upon transfection with prestin. As a result, mechanical perturbations of membranes with prestin cause the generation of an electrical charge transferred through part of or the whole membrane. Here we simulate several mechanical scenarios of charge generation and analyze the charge time-course using our model.

### Electric Charge Resulting from Pressure Step-wise Application

**Figure 6:**
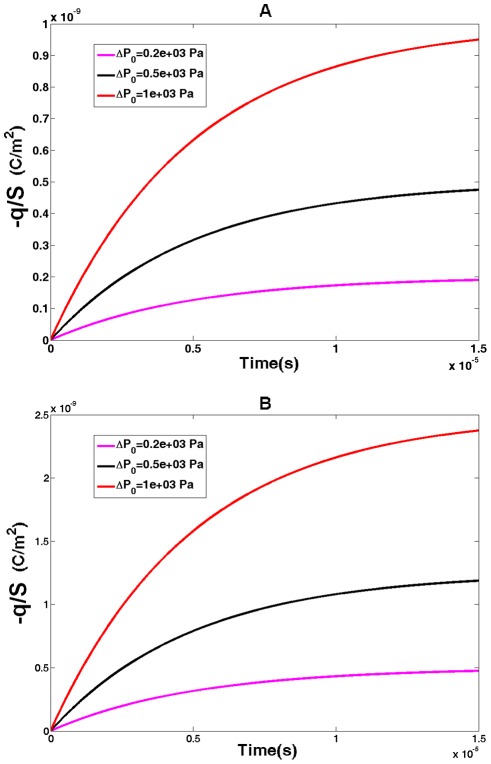
Pressure-generated electric charge I. Charge per unit area (magnitude) generated in cellular membranes with prestin as a result of step-wise application of internal pressure. Three color lines correspond to three values of applied pressure, 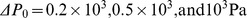. A) outer hair cell and b) cell transfected with prestin.

Here we assume that pressure, ΔP_0_, applied at moment of time, t = 0, is kept constant thereafter. We also assume that the membrane potential is under control (voltage clamp), and it does not change during the time of charge building. The resulting charge is described by Eq. 24 (for outer hair cells) and Eq. 25 (cells transfected with prestin) below. The time course of the charge, q, is presented in Fig. 6 for three magnitudes of the applied pressure, with panel A presenting the outer hair cell case, and panel B showing the case of cells transfected with prestin. The charge clearly evolves toward its steady state value with 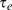 (prestin “RC” time, see the Methods section below) being the characteristic time of the charge change. The amount of charge increases with the applied pressure, and the charge in a cell transfected with prestin (assumed being spherical) is greater than that in the cylindrical outer hair cell of the same radius.

### Electric Charge Resulting from Pressure Constant-Rate Application

**Figure 7:**
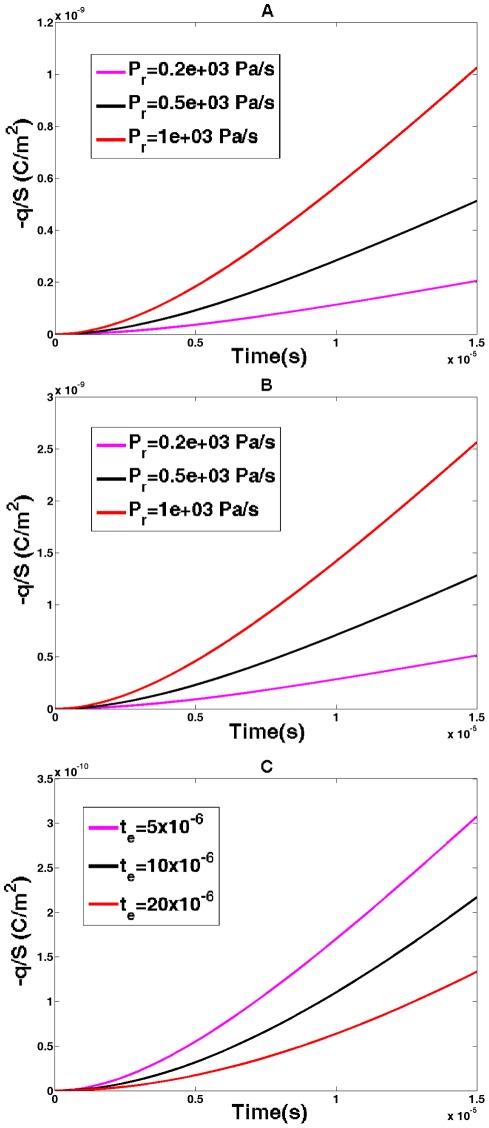
Pressure-generated electric charge II. Charge per unit area (magnitude) generated in cellular membranes with prestin as a result of constant-rate application of internal pressure. A) Outer hair cell for pressure rates of 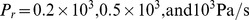, B) cell transfected with prestin for pressure rates of 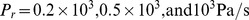, and C) outer hair cell for a fixed-rate pressure and three prestin-associated times of 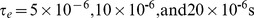.

We now compute the time course of the charge resulting from pressure applied at a constant rate of P_r_. As in the previous case, we assume the membrane potential to be fixed. The resulting charge is obtained from Eqs. 26 and 27 for outer hair cells and cells transfected with prestin, respectively. The computed results are shown in Fig. 7. This figure presents the data for three different pressure rates with panel A representing the outer hair cell case and panel B corresponding to the case of cells transfected with prestin. In both cases, the charge increases with the magnitude of the pressure rate. Also the charge is greater in the cell transfected with prestin than that in the outer hair cell of the equal radius. Since the prestin-associated time,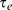, enters both the exponential and linear terms in Eqs. 26 and 27, it is interesting to consider the charge dependence on this parameter beyond its optimal (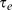 = 5 μs) value. The results show that the charge is smaller for greater prestin-related times (Panel C). All presented curves have a transient parts determined by the exponential terms in Eqs. 26 and 27. For longer times, the time-course converges to a constant-rate (time-linear) regime determined by the first term in parenthesis in Eqs. 26 and 27.

### Electric Charge Resulting from Constant-Rate Strain Application

In the two previous cases of charge transfer resulting from pressure application, the charge evolution was associated with prestin's electrical properties alone. We now consider the condition in which the charge transfer is governed by both the prestin-related electrical properties and viscoelasticity of the membrane. We limit our analysis to the outer hair cell subjected to axial deformation (compression) at a constant rate, 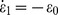. Assuming that the volume of the cylinder (its liquid core) is preserved, the circumferential component of the strain rate is given by the equation 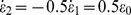. As before, we assume that the cell's membrane potential is fixed during the time course of charge transfer.

**Figure 8:**
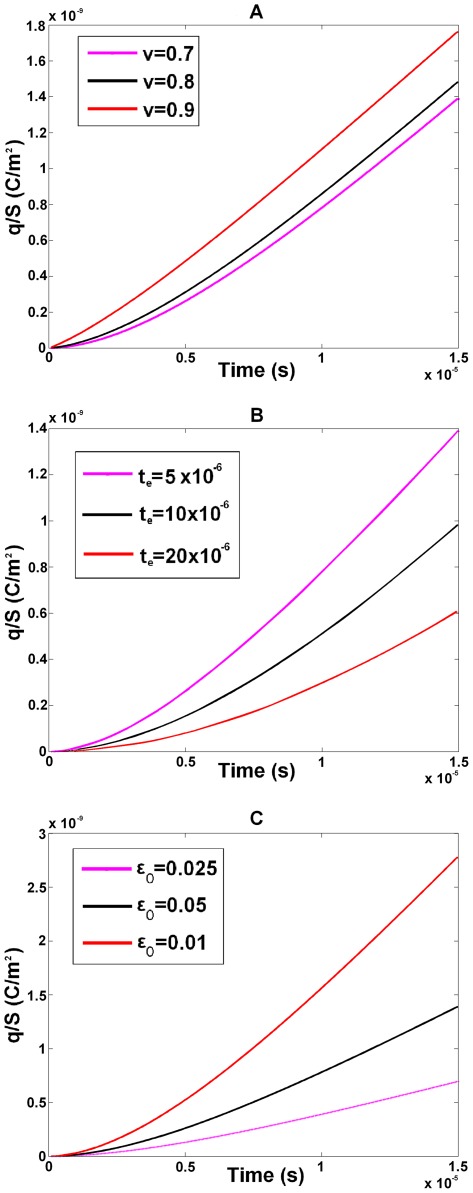
Strain-generated electric charge. Charge per unit area generated in cellular membranes with prestin as a result of constant-rate application of the longitudinal strain to the outer hair cell (with circumferential strain changing accordingly to keep the cell volume constant). A) Three powers entering the viscoelastic model of the cell membrane, 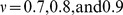, B) three times associated with prestin, 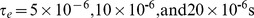, and C) three strains rates 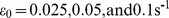.

The results of a parametric study of the charge time course (described by Eqs. 29–31 below) are presented in Fig. 8. In this case, we consider the effects of the electrical and mechanical parameters (beyond their optimal values) as well as the effect of rate of the cell deformation. Panel A shows the charge time course for three different values of the power in our model of membrane viscoelasticity. The lower curve corresponds to the optimal value of the power. The magnitude of the charge increases with the value of the power, ν. Also, when the power, ν, increases and approaches a value of 1, the time dependence of the charge become more linear. Panel B shows the charge dependence on the prestin-associated time, 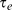. A longer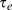-time results in a longer transient regime and smaller magnitudes of the charge. Panel C presents the results for three different values of the strain rate showing an increase in the charge magnitude for higher strain rates. Overall, the curves in Fig. 8 show that charge transfer has two, transient (nonlinear) and constant-rate (linear) regimes determined by the exponential and time-linear terms in Eq. 29. The transient process depends on the prestin-associated time,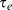, and is modulated by the membrane viscoelasticity. In the case under consideration, the charge is positive in contrast to the previously considered cases of prescribed pressure. This difference is explained by different resultant-strain state of the cell in the regime of the prescribed strain vs. that of prescribed pressure: in the first case, the longitudinal strain and resultant are negative while in the second case they are positive.

### Novel Model of Electrically-Active Viscoelastic Membranes

Here we propose a model that effectively describes cellular electro-viscoleastic membranes operating in a uniquely broad frequency range. The model is derived from first thermodynamic principles. The presented version is developed in the case of cylindrical and spherical cells meaning applications to cells containing the high-frequency protein prestin but it can be extended to a 3-D geometry and can include other electromechanical modes such as bending and curvature. The model allows derivation of a variety of electromechanical characteristics of the cells. It can be effectively applied to analyses of the performance of outer hair cells in vivo as well as under variety of experimental conditions where an electric field, mechanical force, strain, or pressure are applied to the cell in different fashion, including step-wise and constant-rate regimes. The model has explained complex features of such cells, including the active force of almost constant amplitude and phase up to tens of kHz that reveals an unexpected role of the membrane viscoelasticity in boosting the force and balancing the electrical filtering. The power law viscoelasticity plays an important role in characterization of cells under different physiologic (pathological) conditions and time scales (frequency ranges), and the developed model extends this approach to electrically active cells performing at extremely high frequencies. Finally, the obtained constitutive relations generalize the linear piezoelectric relationships with the force and electric field as the independent variables.

## Methods

### Model Derivation from Thermodynamic Principles

In our model derivation, we consider pairs (voltage, V and resultants, N_i_) and (charge, q and strain,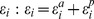) as the independent and dependent variables, respectively. The constitutive relation between the two pairs of variables can be derived in terms of Gibbs free energy, G. As for the purely viscoelastic 3-D case [40], we combine the energy balance equation with the entropy production inequality and neglect time changes and spatial gradients of the temperature. As a result, we obtain the following inequality.
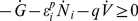
(1)


The first, second, and third terms on the left-hand side of Inequality 1 are the rates of the internal energy, mechanical work (product of the passive component of the strain and resultant) and electrical work inside the membrane, respectively (i = 1,2 and the summation over i-subscript is assumed). The derived inequality is valid for arbitrary independent variables, (N_1_, N_2_) and V. Similar to the methodology of viscoelasticity [40], we assume that the Gibbs free energy is a functional of the history of dependent variables.
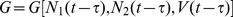
(2)where t and τ are the present and past moments of time, respectively. Then we expand the free energy up to quadratic terms and substitute the result into Inequality 1. The quadratic terms in that expansion can be presented as the products of linear combinations of the dependent and independent variables multiplied by arbitrary rates of the dependent variables. In order Inequality 1 to be valid for such arbitrary independent variables, these linear combinations of the independent and dependent variables must be equal to zero, which results in the following constitutive relations



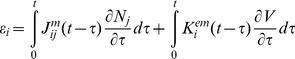
(3)

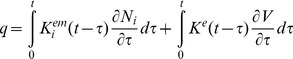
(4)where summation over the j-subscript on the right-hand side of Eqs. 3 and 4 is assumed. The coefficients (kernels) 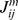, 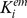, and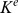 are determined by the dependence of the free energy on its arguments (Eq. 2), and they represent, respectively, the mechanical, electromechanical, and electrical properties of the membrane. These kernels will be discussed in detail below. The full derivation of the electro-viscoelalstic constitutive relations is not included since it follows that in the case of pure viscoelasticity [40], [41]. Also note that Eqs. 3 and 4 present a generalization of linear piezoelectric relationships with the force and electric field as the independent variables (e.g., [42]).

### Electrical and Electromechanical Kernels

In terms of the electrical kernel in constitutive Eq. 5, we show that the electrical properties of prestin, including frequency dependence, resistive component of the charge, and its phase shift found earlier ([30]) can be well described by a combination of a capacitor and resister connected in series (see below). The corresponding electrical kernel that relates the charge per unit area and voltage is expressed as
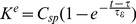
(5)where C_sp_ is the specific capacitance of the membrane and τ_e_ is typical (RC) time associated with prestin charge transfer. We also interpret the mechanical and electrical components of the strain (first and second term on the right-hand side of Eq. 3) as its passive and active parts, respectively. The portion of the transferred charge associated with the electric field is described by the electrical kernel, K^e^, in Eq. 4. Since the active strain and the transferred charge are tightly connected, we choose the electromechanical kernels to be proportional to the electrical kernel:
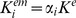
(6)where αi are the coefficients that are estimated below. The resulting constitutive relations can be now presented as



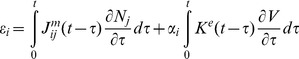
(7)

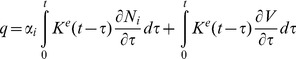
(8)


In the case of cells transfected with prestin whose membrane is assumed to be spherical and isotropic, the constitutive equations take a slightly different form:
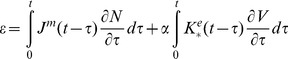
(9)

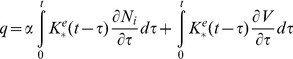
(10)Here (as in the case of the constitutive relations of isotropic viscoelasticity [40], [41]) ε and N are, respectively, the isotropic parts of the strain and resultant tensors. Also, J^m^ and K^e^ are the mechanical and electrical kernels, respectively, and α is a single coefficient of proportionality between the electromechanical and electrical kernels.

### Mechanical Kernel

As has been done in recent viscoelastic models of cells, we assume a power law relation between the mechanical variables. Traditionally, two regimes, creep and relaxation, are used for the characterization of a viscoelastic material with the notations of the corresponding kernels as *J* and *E.* Thus in Eq. 3, where the resultants and strain are, respectively, the independent and dependent variables (like in creep regimes), we use *J*-notation for our mechanical kernels. To derive the mechanical kernel, we start with the outer hair cell case. We first introduce the corresponding relaxation kernels that take the following form
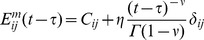
(11)where C_ij_ are the elastic moduli of the outer hair cell orthotropic composite membrane, ν and η are parameters of the viscous part of the kernel estimated below, and Γ is the gamma-function. Then the corresponding 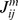
- kernels can be determined in terms of their Laplace transform. The matrix with components 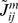 is given by the following equation



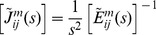
(12)where the components of the matrix on the right hand side of Eq. 12 can be explicitly written as
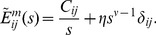
(13)


The relaxation functions will also be introduced in the frequency domain (see Eq. 18 below).

In the case of cells transfected with prestin, the mechanical (creep) kernel, *J^m^*, is associated with the relaxation kernel, *E*, via the same equation as that for the outer hair cell case considered above where the relaxation kernel takes the following form
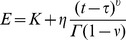
(14)where *K* is the area modulus of the isotropic spherical membrane.

### Approximation of the Fokker-Planck Equation for Prestin-Associated Charge Transfer

**Figure 9:**
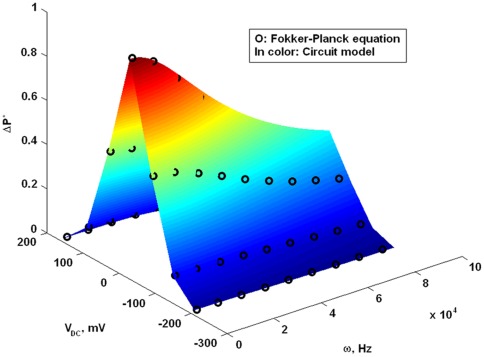
Analytical description of prestin-associated charge. Approximation of the solution of the Fokker-Planck equation obtained in [30] (circles) by an electrical filter function (color surface) for different DC-potentials, V_DC_, and frequencies, ω.

Previously [30], we introduced a Fokker-Planck equation in which the prestin-associated charge was described in terms of the total probability, ΔP^*^, of charge transfer. The time and frequency dependence of this function can be well approximated by the solution of the following ODE
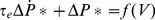
(15)where τ_e_ is the typical time of electric charge transfer by prestin, and *f* is a function of the applied voltage obtained from the solution of the Fokker-Planck equation. In the case of a harmonic (DC+AC) voltage, 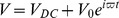, the time and frequency dependence of ΔP^*^ is described as



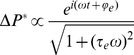
(16)where
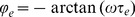
(17)is a phase shift determined by the process of charge transfer by prestin. Fig. 9 presents an approximation of the solution of the Fokker-Planck equation in terms of the 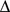
P^*^-amplitude obtained by using the frequency function in Eq. 16. The best fitting surface presented in Fig. 9 (a function of ω and V_DC_) corresponds to the time parameter, τ_e_ = 5μs.

**Figure 10:**
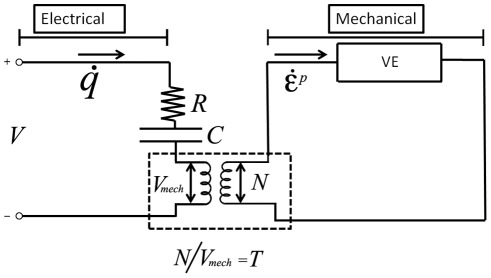
Circuit analog of the constitutive equations. The left panel represents the electrical part of the equations where V, R, C, and 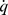 are, respectively, the membrane voltage, resistance and capacitance associated with the charge transferred by prestin, and the rate of the transferred charge (current). The right panel represents the mechanical part of the model equations, where the membrane resultant, N, and the rate of passive strain, 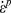, play the role of the voltage and current, respectively. The VE-box represents the viscoelastic transformation between the resultant, passive strain, and strain rate. The low central panel with the transformer (two coils) represents the electromechanical coupling with transformer ratio T between the resultant, N, and the mechanical component of the voltage, V_mech_.

The analytical description of prestin-related charge transfer (Eqs. 15 and 16) is equivalent to a simple circuit in which a capacitive, C, and resistive, R, elements are connected in series, and the product, RC, is equal to the time parameter, τ_e_. The Fokker-Planck equation in [30], its analytical approximation (Eq. 15), and the equivalent RC-circuit describe the prestin-associated charge transfer under the action of purely electric field with no effect of coupling to the mechanics of the membrane. In order to have an electrical representation of the whole model of electro-viscoelastic membranes, the prestin-related circuit has to be coupled to another circuit representing the passive viscoelastic properties of the membrane (Fig. 10). The electromechanical coupling in Fig. 10 is represented by an ideal transformer (two coils) with the transformer ratio, T, between the voltages in the left and right circuits [24], [26], [43], [44]. In the right circuit, the voltage and current have the meaning of the mechanical resultant, N, and rate of the passive strain, 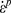, respectively. The VE-box in the right circuit represents the viscoelastic relationship between the membrane resultant, passive strain, and the rate of the passive strain. The passive strain is the difference between the total and active strains. The active strain (consistent with our treatment of the electromechanical kernel in Eq. 6) can be considered being proportional to the membrane voltage V_,_ in the left circuit. The viscoelastic component balances the electrical one in the frequency dependence of isometric (corresponding to 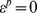) membrane resultant illustrating the frequency-invariance of the outer hair cell isometric force in our analytical model. Note that the circuit consideration is not fully equivalent to the original two-dimensional elastically-orthotropic model, and it illustrates in a simpler way the main features of the model.

### Derivation of Membrane Electromechanical Characteristics

#### Active Force Generated by a Harmonic Electric Field

We transform the constitutive relation to harmonic variables. It can be shown that the mechanical kernel, 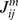, in the time domain corresponds to the following complex modulus, 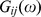, in the Fourier domain:
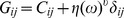
(18)


This modulus relates the harmonic resultants and passive strains:
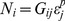
(19)


Because we are assuming isometric conditions, we substitute 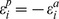 and transform Eq. 19 into the following system of equations
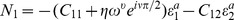
(20)

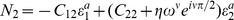



Similar to our analysis in the time domain, we assume that the active strain is proportional (with the coefficients, α_i_) to the transferred charge whose amplitude, q_0,_ is given by
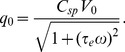
(21)


Here V_0_ is the amplitude of the applied voltage. Finally, the amplitude (normalized by its value at low frequencies) is given by the equation
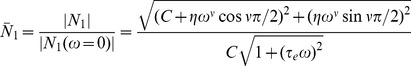
(22)where 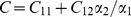. The force phase is described by the following equation


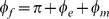
 where 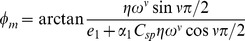
(23)where 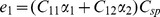 and the prestin-associated phase shift, 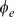, is given above by Eq. 17.

#### Electric Charge Resulting from Mechanical Perturbations

To derive the charge resulting from pressure step-wise application we use Eqs. 8 and 10 with no voltage terms. The time course of the building charge is described by the following equations
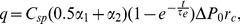
(24)in the case of the outer hair cell and



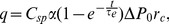
(25)in the case of spherical cells transfected with prestin. In both cases, r_c_ is the cell radius.

We now derive the time course of the charge resulting from pressure applied at a constant rate of P_r_. As in the previous case, the voltage terms in the constitutive equations can be dropped. We substitute constant τ-derivatives of the resultants in Eqs. 8 and 10 and do the integration over τ. As a result, we obtain
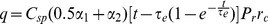
(26)


and
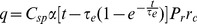
(27)for the outer hair cells and cells transfected with prestin, respectively.

Finally we derive the time course of the charge resulting from an axial constant-rate deformation of an incompressible cylindrical outer hair cell. In order to use the constitutive relation for the charge (Eq. 8), we first have to compute the rates of the resultants. The resultant rates can be expressed in terms of the prescribed strain rates by using the relaxation kernels introduced in Eq. 7. As a result of this derivation, we obtain
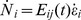
(28)


Then, the resultant rates are substituted into Eq. 8, assuming that the voltage-related term is dropped. After this substitution, the integration in Eq. 8 is developed partially analytically and partially numerically. The resulting equation for the charge takes the following form
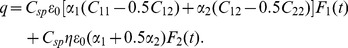
(29)


Here the functions, 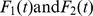 are given by the equations
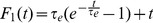
(30)


and
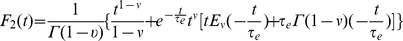
(31)


Function *E_υ_* in Eq. 31 is the exponential integral defined as
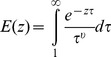



This function was evaluated numerically by using Wolfram Mathematica.

#### Summary of Model Parameters

The mechanical parameters of the model include the elastic moduli, C_11_, C_12_, and C_22_, of the outer hair cell wall, area modulus, K, of the plasma membrane of cells transfected with prestin, and viscoelastic parameters, η and υ, assumed to be the same for both types of cells. The C_ij_-moduli have previously been estimated [27], [37], [45]. Here we use the following values
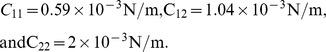
The modulus, K, was previously estimated as [46]K = 0.24 N/m.

Finally, the above estimated viscoelastic parameters (section Results and Discussion) are
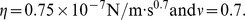



The main electrical parameter of the model is the typical time of charge transfer by prestin, τ_e_. The prestin-associated time was estimated above by approximating the solution of Fokker-Panck equation, which resulted in
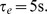



Finally, the electromechanical parameters of the model are the constants, α_i_ (Eqs. 7 and 8 in the outer hair cell case) and α (Eqs. 9 and 10 in the case of cells transfected with prestin). Taking into account Eq. 5 for the electrical kernel, the electromechanical parameters enter the constitutive equations via the products, 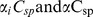. These products can be found by using the previously made estimates of the steady state (when 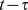
>>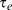) values of the active strain, 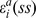. In the case of outer hair cell, the relationship between the two is given by the equations


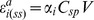
 or 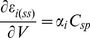


The steady state active strain and its voltage derivatives can be extracted from the electromotile length and radius changes obtained in the microchamber experiment [47], [48], which results in the following estimates of the electromechanical parameters [27].


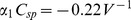
 and 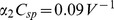


Similar estimates can be obtained in the case of cells transfected with prestin. The data for HEK cells [17] show that changes in the transmembrane potential of about 0.4 V result in a displacement of about −0.1 μ. Assuming the cell radius to be about 5 μ, we obtain